# Anxiety symptoms and coping strategies in the perinatal period

**DOI:** 10.1186/1471-2393-13-233

**Published:** 2013-12-13

**Authors:** Astrid George, Rita F Luz, Claude De Tychey, Nathalie Thilly, Elisabeth Spitz

**Affiliations:** 1 Department of Psychology, Laboratory EA 4360 Apemac-EPSaM, University of Lorraine, Ile du Saulcy BP 30309 57006 METZ, Cedex 1, France; 2 Department of Psychology, Laboratory Interpsy, EA 4432, University of Lorraine, Nancy, France; 3 Department of Epidemiology and Clinical Evaluation, CIC-EC CIE6 Inserm, Academic Medical Centre, Brabois, Nancy, France; 4 University of Lorraine EA 4360 Apemac Nancy, 9 Avenue de la Forêt de Haye, 54500 Vandoeuvre-lès-Nancy, France

**Keywords:** Childbirth, Anxiety, Coping, Ante- and postnatal

## Abstract

**Background:**

The aim of the present study was to explore the prospective relationship between anxiety symptoms and coping strategies during late pregnancy and early postpartum.

**Methods:**

Participants completed the Hospital Anxiety Depression-Anxiety subscale and Carver’s Brief COPE at two time points, namely during the last trimester of pregnancy (N = 400) and at two months postpartum (N = 158).

**Results:**

Antenatally, 18.8% of pregnant women presented severe anxiety symptoms while 20.2% of women presented severe anxiety symptoms after birth. Carver's proposed coping styles allowed to significantly distinguish between anxious and non anxious women during these two periods. Anxious women used significantly more inappropriate coping and less adaptive coping responses, such as self-blame and denial of reality, which remained associated with anxiety in the perinatal period. Our results also indicated a decrease in adaptive coping in women without anxiety after birth (e.g. acceptance, positive reframing).

**Conclusion:**

Our findings confirm that antenatal and postnatal anxiety symptoms occur frequently and that inappropriate and/or non functional coping may account for persisting anxiety after childbirth. Limitations: Data were based on self-reports and participating women were predominantly primiparous. A high drop-out rate at two months postpartum must also be acknowledged.

## Background

Pregnancy and postpartum periods are known as sensitive periods in a woman’s life. This specific time-frame, encompasses a major risk of psychiatric morbidity [1]. However, there remains a lack of longitudinal studies regarding psychological distress and development of mental illness, even though this issue represents a major public health concern for women and their children. Anxiety symptoms in the perinatal period are frequent [2]. Nonetheless, data pertaining to prevalence rates of anxiety disorders are limited and are impeded by a number of factors including a deficit in research [3], the definition of anxiety during pregnancy and postnatal period as a specific category [4] and a heterogeneous use of psychometric data [5]. Moreover, much of the published research has only assessed postpartum anxiety through retrospective chart reviews or cases studies, thus making it difficult to draw acceptable conclusions as to the prevalence of perinatal anxiety and the relationship between prenatal and postnatal anxiety.

Study data also highlight that severe psychological stress such as anxiety during pregnancy, fear of childbirth and anxiety after childbirth, is associated with exposure to obstetrical intervention as well as with negative neonatal outcome for the mother and infant [6]. Indeed, studies have shown that women with high distress in late pregnancy are more likely to develop postpartum depression [7] and that the development of the foetus and the child is negatively influenced by stress and anxiety during pregnancy [8-10]. Moreover, negative neonatal outcome including prematurity and low birth weight has been associated with pregnancy anxiety [11,12]. Additionally, the occurrence of anxiety and subthreshold anxiety symptoms can be detrimental to the relationship between the mother and her infant [13]. Hence, current research needs to focus on psychological adaptation to prevent mental illness, especially during the perinatal period. Prevention is thus an important tool in research and clinical application. In keeping with view, coping strategies have been widely investigated to understand the relationship between psychological stress and the human capacity to deal with challenges [14]. Giving birth is a major life event; thus managing the issues of pregnancy and the transition to motherhood call for psychological adjustment [15].

The aims of the present study were: (1) to evaluate the prevalence of prenatal and postnatal anxiety symptoms in women living in Lorraine, and (2) to study coping strategies in women with anxiety symptoms in the prenatal and/or postnatal period compared to women showing low or no anxiety.

Our hypothesis is that women with adaptive coping show less or no anxiety symptoms whereas anxious women will show inappropriate coping. Highlighting the relationship between adaptive coping and low anxiety appears instrumental in preventing anxiety and negative outcomes for both mother and child. Our goal is to discern different coping responses within these groups to emphasize the relationship between adaptive coping and well-being.

## Methods

### Sample

We followed 400 French women during their pregnancy from the 26^th^ - 35^th^ week of gestation (prenatal phase evaluation - T1) to 6–8 weeks after childbirth (postnatal phase evaluation - T2). Of these, 173 completed and returned all questionnaires along with their written consent in both the pre– and postnatal periods; however, 15 women had missing data with regard to the *Brief Cope* questionnaire and were excluded from time and group effects, bringing the total number of responders to 158 for the postnatal evaluation. The investigation and aim of the study were presented to study participants during birth preparation classes. The medical personnel agreed to recruit the women and to take part in the research.

Ethical approval was obtained for the study (*Comité de protection des personnes Est-II, protocol n°10/561*).

### Procedure

The study was presented to the women during birth preparation classes at the hospital or during a midwifery consultation. Each woman completed the questionnaire including the HAD-A Subscale, the Brief COPE questionnaire and demographic information. Women completed the questionnaire at home and returned the latter to the investigative group at the University in accompanying postage-prepaid envelopes and gave written consent to participation in this study.

After the second evaluation, every woman received written information regarding psychological assistance, regardless of her anxiety score before or after delivery, in order to help those women with psychological distress. Processing of data and statistical analyses were performed by personnel other than those collecting the data.

### Measurements

#### Anxiety

In the prenatal (T1) and postnatal phases (T2), the women’s anxiety level was measured using the self-administered HADS-A questionnaire [16]. The HADS-A is a seven-item anxiety subscale assessing the presence or absence of anxiety symptoms over the past week on a four-point scale for each item (0–3). Total score ranged from 0 to 21.

For analysis purposes, anxiety level was classified into three groups: 'no anxiety’ for a score of 0–7, 'moderate anxiety’ for a score of 8–10, and 'severe anxiety’ for a score greater than 10 [17]. In accordance with current literature, cut-off values of 8 and 10 were respectively chosen to maximize sensitivity and specificity [18].

#### Coping

Psychological coping was evaluated using the Brief COPE [19] in its French version [20]. The Brief COPE is a 28-item questionnaire allowing to distinguish 14 distinct coping strategies when confronted with a stressful event. Each item is assessed on a 0–4 point Likert scale. The 14 conceptually differentiable coping reactions are: active coping, planning, using emotional support, using instrumental support, venting, positive reframing, acceptance, denial, self-blame, humour, religion, self-distraction, substance use, behavioural disengagement. Some of these reactions are known to be generally adaptive; others are known to be problematic. Two items represent one of the 14 coping strategies and scores for each coping strategy range from 0 to 8. Classifying coping strategies into “adaptive and non-adaptive” strategies remains difficult because this evaluation depends on the stressor and the short- and long-term effects. In general, problem-focused coping strategies such as planning, active coping, positive reframing, acceptance and humour are known as adaptive coping strategies, whereas denial, self-blame, distraction and substance use are more often associated with negative emotions such as shame, guilt, lower perception of self-efficacy and psychological distress.

#### Statistical analyses

Continuous variables are presented as means ± standard deviation and categorical variables as percentages. Demographic characteristics were compared between the three anxiety groups using Pearson's chi-squared test and analysis of variance for qualitative and quantitative variables, respectively. The relationship between anxiety before and after birth was estimated by the Kappa coefficient. Analysis of variance models were used to test: (1) the evolution of coping scores between T1 and T2, regardless of the anxiety level (Time effect), (2) the association between anxiety level (no/moderate/severe anxiety) and each coping strategy, regardless of the time of measurement (T1, T2) (Group effect). A P-value of < 0.05 for two-sided tests was considered significant. All analyses were performed with SPSS software version 17.0.

## Results

### Sample characteristics

Recruited women were between 19 to 46 years of age with a mean age of 29.2 ± 4.7 years. The majority of women were primiparous (83.5%), lived with the child’s father (92.5%) and had a planned pregnancy (78.2%). Difficulties in getting pregnant were reported by 30.8% of the women while 27.7% indicated complications during pregnancy. Our postnatal population did not differ from the prenatal sample according to mean age (29.1 years, SD 4.9), primiparous (84.5%), living with the baby’s father (95.2%), difficulties in getting pregnant (29.9%) or complications during birth (25.6%). Before birth, these characteristics did not differ between the three aforementioned anxiety groups (*p* from 0.28 to 0.92). Apparent differences in anxiety observed among the three groups were not attributable to differences in these categories. After birth, there was also no significant relationship, aside from a tendency for age: younger women appeared to be more anxious after birth, although the difference was not statistically significant (*p* = 0.10). In addition, we analysed differences between responders and non-responders within these categories as well as their anxiety score: there was no significant differences between women responding after birth and non-responders.

### Prevalence of prenatal and postnatal anxiety

At the prenatal phase, 18.5% (n = 74/400) of pregnant women presented severe anxiety symptoms while 24.5% (n = 98/400) presented moderate anxiety symptoms. At the postnatal phase, anxiety prevalence was higher with 20.2% (35/173) of women presenting severe anxiety symptoms and 24.3% (N = 42/173) moderate anxiety symptoms. The relationship between anxiety before and anxiety after giving birth was relatively high (Kappa coefficient = 0.46): 59.3% of the women who were anxious at the prenatal phase remained anxious after birth.

Firstly, results show time effects regarding the use of coping strategies before and after giving birth: nearly all coping strategies declined at 2 months postpartum, except for substance use, which increased significantly (p = 0.023), and for humour and behavioural disengagement, which showed no significant differences in pre- and postnatal population (see Table 1).

**Table 1:** Coping strategies according to prenatal and postnatal anxiety levels, including means, standard deviations (SD), F and degrees of freedom as well as comparisons between groups over time

**Coping strategies**	**T1 (3rd trim. pregnancy) n = 400**	**T2 (2 months postpartum) n = 158**	**Group effect**		**Time effect**	
	**Mean (SD)**	**Mean (SD)**	**F(df)**	***p***	**F(df)**	***p***
**Active coping**						
No anxiety	3.15 (0.45)	3.00 (0.65)				
Moderate anxiety	3.09 (0.45)	3.04 (0.48)				
Severe anxiety	3.08 (0.54)	2.97 (0.67)	0.16 (2)	n.s.	4.85 (1)	0.03
**Planning**						
No anxiety	3.12 (0.46)^g^	2.98 (0.61)^g^				
Moderate anxiety	3.15 (0.45)	3.03 (0.47)				
Severe anxiety	3.02(0.46)	3.08 (0.58)	0.13 (2)	n.s.	4.23 (1)	0.04
**Using instrumental support**						
No anxiety	3.15 (0.64)^g^	2.89 (0.81)^g^				
Moderate anxiety	3.09 (0.62)	3.06 (0.70)				
Severe anxiety	3.19 (0.77)	2.95 (0.84)	1.33 (2)	n.s.	10.65 (1)	0.001
**Using emotional support**						
No anxiety	3.17 (0.72)	2.94 (1.23)				
Moderate anxiety	3.27 (0.58)	3.12 (0.65)				
Severe anxiety	3.29 (0.69)^i^	2.94 (0.83)^i^	0.59 (2)	n.s.	6.87 (1)	0.01
**Venting**						
No anxiety	3.11 (0.60)^g^	2.91 (0.72)^g^				
Moderate anxiety	3.03 (0.71)	2.94 (0.64)				
Severe anxiety	3.15 (0.71)	2.92 (0.89)	0.07 (2)	n.s.	9.58 (1)	0.002
**Acceptance**						
No anxiety	3.39 (0.36)^d.e^	3.38 (0.47)				
Moderate anxiety	3.24 (.036)^d^	3.24 (0.58)				
Severe anxiety	3.13 (0.48)^e^	3.32 (0.61)	2.89 (2)	0.05	0.64 (1)	ns
**Denial**						
No anxiety	1.63 (0.75)^a.e.g^	1.43 (0.59)^a.b.g^				
Moderate anxiety	1.92 (0.72)^a^	1.68 (0.76)^a^				
Severe anxiety	1.90 (0.69)^e^	1.81 (0.85)^b^	5.44 (2)	0.05	7.87 (1)	0.06
**Self-Blame**						
No anxiety	2.31 (0.75)^a.g^	1.98 (0.69)^a.b.g^				
Moderate anxiety	2.44 (0.70)^b^	2.33 (0.81)^a^				
Severe anxiety	2.87 (0.84)^a.b^	2.63 (0.90)^b^	12.64 (2)	0.001	11.23 (1)	0.001
**Humour**						
No anxiety	1.97 (0.80)	1.98 (0.85)				
Moderate anxiety	1.78 (0.67)	1.72 (0.73)				
Severe anxiety	1.73 (0.63)	1.68 (0.65)	3.19 (2)	0.04	0.07 (1)	n.s.
**Religion**						
No anxiety	1.90 (0.94)^g^	1.70 (0.91)^g^				
Moderate anxiety	1.63 (0.81)	1.58 (0.79)				
Severe anxiety	1.90 (0.92)^i^	1.60 (0.93)^i^	0.779 (2)	n.s.	9.43 (1)	0.01
**Distraction**						
No anxiety	2.88 (0.74)^g^	2.37 (0.93)^g^				
Moderate anxiety	3.04 (0.59)^h^	2.46 (0.74)^h^				
Severe anxiety	2.73 (0.70)^i^	2.19 (0.92)^i^	1.79 (2)	n.s.	49.72 (1)	0.001
**Substance use**						
No anxiety	1.05 ( 0.18)	1.13 (0.45)				
Moderate anxiety	1.10 (0.28)	1.12 (0.29)				
Severe anxiety	1.03 (0.18)	1.26 (0.68)	0.56 (2)	n.s.	5.275 (1)	0.023
**Behavioural Disengagement**						
No anxiety	1.51 (0.49)	1.50 (0.59)				
Moderate anxiety	1.77 (0.68)	1.65 (0.61)				
Severe anxiety	1.61 (0.57)	1.58 (0.65)	2.81 (2)	n.s.	0.50 (1)	n.s.
**Positive reframing**						
No anxiety	3.01 (0.60)^d^	2.93 (0.58)^a^				
Moderate anxiety	2.91 (0.48)	2.77 (0.69)				
Severe anxiety	2.73 (0.70)^d^	2.60 (0.69)^a^	4.22 (2)	0.016	3.71 (1)	ns

Note: Group effect: ^a,b,c^*: p* ≤0.05; ^d,e,f^: 0.05 > *p* < 0.10; n.s.: non significant.

Time effect: ^g,h,i^*: p* ≤0.05.

Secondly, regarding group effects, in the last trimester of pregnancy, the women presenting severe anxiety symptoms used coping strategies significantly less often with adaptive values such as acceptance (*p* = 0.016), positive reframing (*p* = 0.025) and humour (*p* = 0.04) than women presenting moderate or no anxiety (see Table 1). Additionally, the higher the level of anxiety, the more likely the women used problematic coping responses such as denial (*p* = 0.05) and self-blame (*p* = 0.001).

At the postpartum phase, severe anxiety was associated with the self-blame strategy of coping (*p ≤0.05*) and women with severe anxiety were more likely to use denial (*p ≤*0.05). Moreover, two months after birth, women with severe anxiety had significantly more difficulties in positive reframing of their new situation than women presenting no anxiety.

Lastly, results showed no interaction effect between time and group effect.

## Discussion

The results of the present study highlight the fact that perinatal anxiety is a public health problem: 18.8% of the women in our sample presented severe anxious symptoms prior to birth and 20.2% after birth. This indicates that the prevalence of prenatal anxiety is as important as that for depression, although it would appear that after birth, anxiety prevalence is twice as high as postnatal depression in a similar French population [21]. Skouteris et al. [7] proposed a bi-directional model of depression and anxiety, which could be brought into account in the present instance since depression was not controlled in our study. While we are well aware that general anxiety measurements may have limited validation in specific populations [22], our results nevertheless indicate the stability of pre- and postnatal anxiety. These findings are in accordance with observations reported in the literature [23]. Furthermore, studies have shown that women differ with regard to the nature and timing of heightened anxiety during the transition to motherhood [24]. Therefore, specific measures for anxiety in the perinatal period are strongly recommended [4,5]. Moreover, existing measurements must be translated and validated in French in order to address these potential differences in our French population.

Of particular interest, it seemed somewhat surprising that anxious and non anxious women did not differ with regard to their demographic characteristics. We presume that in late pregnancy, anxiety may be more related to fear of childbirth, with the infant’s health being a key preoccupying concern. Such information could be enhanced though clinical interviews in order to improve the quality of knowledge.

The high number of women reporting having difficulties in getting pregnant or complications during pregnancy must be interpreted with caution since self-reported measurements were used. Objective measures are necessary to properly evaluate the timeframe prior to the women becoming pregnant and the severity of complications during pregnancy. Such medical information could then be compared with the women’s perceptions regarding these two issues.

In accordance with our hypothesis, anxious women used significantly more inappropriate coping and less adaptive coping responses during late pregnancy. Although self-blame (criticizing oneself for responsibility in the situation) and denial (trying to push the reality of the situation away) remained associated with anxiety before and after birth, we found no persistent results regarding adaptive coping strategies. Our data rather indicate a decrease in adaptive coping in women without anxiety after birth (e.g. active coping, positive reframing). One must take into consideration that adaptation to maternity is influenced by numerous factors which were not entered into account in the present instance, such as maternal orientations [24], self-perceived distress [25] and depression [12].

There are several clinical implications of our findings, including prevention of postnatal distress by improving adaptive coping such as positive reframing and acceptation, which appear to be especially employed by women without clinical anxiety. Prevention implies screening for anxiety levels in women at the beginning of the prenatal period and to offer therapeutic support as soon as possible for women presenting high anxiety levels. Another possible means to improve the adaptive coping strategies for anxious women might be to conduct postpartum groups to answer questions and to learn positive reframing. Such improvement would represent a highly useful attribute during the perinatal period, because of the developmental and environmental changes to which women are exposed to.

Several limitations should be considered. Firstly, this study features an important dropout rate, in part certainly due to the fact that the birth of a baby brings about substantial changes in the women’s lives and claims for reorganisation. In addition, several families moved before the birth and left no address.

Second, only self-reporting data was used. Moreover, women were recruited from birth preparation classes thus resulting in a likely selection bias: overrepresentation of primiparous pregnancies and without major complications requiring special medical monitoring during pregnancy. Our choice of assessment during the third trimester of pregnancy can be justified for several reasons: the increasing level of anxiety during the course of pregnancy, the fact that most interrupted pregnancies occur during the first 16 weeks and that most of the women had completed their prenatal diagnosis before the third semester. In the case of the postnatal period, the choice of two months after delivery for retesting is in keeping with current postnatal research.

Altogether, we believe our results offer new clinical implications and a perspective for further research regarding perinatal anxiety. A better understanding of anxiety in this sensitive period in both the women's and infant’s lives would allow the possibility of developing better preventive strategies towards improving mother-baby interaction, and avoiding negative outcomes.

## Conclusion

In conclusion, our results show that pre- and postnatal anxiety is a major health issue and that severe anxiety during pregnancy and after childbirth is associated with less adaptive coping. Denial and self-blame as problematic choices to deal with stress are persistently used by anxious women over time. It appears important to gain further knowledge regarding the long term effects of anxiety and coping strategies on women, and whether their coping strategies subsequently increase thereafter.

## Competing interests

The authors report no conflict of interests.

## Authors’ contributions

AG and ES conceived the study. AG carried out the study and drafted the manuscript AG is the main author and carried out the study, ES participated in its design and coordination and helped to draft the manuscript. NT performed the statistical analysis, RL and CT helped to draft the manuscript. All authors read and approved the final manuscript.

## Pre-publication history

The pre-publication history for this paper can be accessed here:

http://www.biomedcentral.com/1471-2393/13/233/prepub
